# EFFECT OF RISE ON REPEAT ELDER ABUSE AND SELF-NEGLECT INVESTIGATIONS BY ADULT PROTECTIVE SERVICES

**DOI:** 10.1093/geroni/igad104.1156

**Published:** 2023-12-21

**Authors:** Stuart Lewis, Andie MacNeil, M T Connolly, Erin Salvo, Patricia Kimball, Geoff Rogers, David Burnes

**Affiliations:** Dartmouth College, Lebanon, New Hampshire, United States; University of Toronto, Toronto, Ontario, Canada; University of Southern California, Los Angeles, California, United States; Department of Health and Human Services, Augusta, Maine, United States; Elder Abuse Institute of Maine, Brunswick, Maine, United States; City University of New York, New York City, New York, United States; University of Toronto, Toronto, Ontario, Canada

## Abstract

Adult Protective Services (APS) is the primary agency responsible for investigating Elder Abuse and Self-Neglect (EASN) allegations in the US. The harms of EASN are well established; however, APS lacks a conceptually derived, evidenced-based intervention phase. RISE is a community-based EASN intervention designed to complement and augment APS that provides enhanced services and a longer intervention phase. The objective of this study was to test whether exposure to the APS/RISE collaboration reduced the case outcome of recurrence (repeat investigations) compared to usual APS only services. This study was based on a retrospective observational design (n = 1947) in two randomly selected counties of Maine where RISE was available to provide enhanced services to persons referred from APS. Analysis used an extended regression endogenous treatment Probit model using APS administrative data to predict case recurrence. Between 2019 and 2021, 154 cases participated in RISE and 1793 received usual APS only services. 49% of cases referred to RISE had a complex history with 2 or more prior substantiated allegations versus 6% for those receiving usual APS only services. After accounting for the non-random treatment assignment, RISE significantly lowered the likelihood of recurrence compared to persons receiving usual care provided by APS (probability of recurrence reduced by 0.55 for the Average Treatment Effect on the Treated and 0.26 for the Average Treatment Effect). A reduction in recurrence carries important implications for APS costs, resources, and workflow. It may also serve as a proxy indicating a reduction in re-victimization and harm for EASN victims.

